# Utility of a rapid assay for prostaglandin E-major urinary metabolite as a biomarker in pediatric ulcerative colitis

**DOI:** 10.1038/s41598-023-37145-6

**Published:** 2023-06-19

**Authors:** Shin-ichiro Hagiwara, Naoki Abe, Kenji Hosoi, Tomoko Hara, Takashi Ishige, Hirotaka Shimizu, Tatsuki Mizuochi, Toshihiko Kakiuchi, Reiko Kunisaki, Ryo Matsuoka, Hiroki Kondou, Fumihiko Kakuta, Yoshiko Nakayama, Takeshi Kimura, Takatoshi Maeyama, Hitoshi Honma, Daishi Hirano, Masayuki Saruta, Tsutomu Yoshida, Isao Okayasu, Yuri Etani

**Affiliations:** 1 Department of Gastroenterology, Nutrition, and Endocrinology, Osaka Women’s and Children’s Hospital, 840 Murodocho, Izumi, Osaka 594-1101 Japan; 2 Division of Infectious Diseases and Immunology, Aichi Children’s Health and Medical Center, Aichi, Japan; 3 Department of Gastroenterology, Tokyo Metropolitan Children’s Medical Center, Tokyo, Japan; 4 Department of Gastroenterology and Hepatology, Saitama Children’s Medical Center, Saitama, Japan; 5 Department of Pediatrics, Gunma University Graduate School of Medicine, Maebashi, Japan; 6 Center for Pediatric Inflammatory Bowel Disease, Division of Gastroenterology, National Center for Child Health and Development, Tokyo, Japan; 7 Department of Pediatrics and Adolescent Medicine, Juntendo University Graduate School of Medicine, Tokyo, Japan; 8 Department of Pediatrics and Child Health, Kurume University School of Medicine, Kurume, Japan; 9 Department of Pediatrics, Faculty of Medicine, Saga University, Saga, Japan; 10 Inflammatory Bowel Disease Center, Yokohama City University Medical Center, Yokohama, Japan; 11 Department of Pediatrics, Fuji City Central Hospital, Fuji, Japan; 12 Department of Pediatrics, Kindai University Nara Hospital, Nara, Japan; 13 Department of General Pediatrics and Gastroenterology, Miyagi Children’s Hospital, Sendai, Japan; 14 Department of Pediatrics, Shinshu University School of Medicine, Matsumoto, Japan; 15 Department of Pediatrics, Osaka University Graduate School of Medicine, Osaka, Japan; 16 Department of Pediatrics, The Jikei University School of Medicine, Tokyo, Japan; 17 Division of Gastroenterology and Hepatology, Department of Internal Medicine, The Jikei University School of Medicine, Tokyo, Japan; 18 Division of Molecular Pathology, Department of Comprehensive Medicine, Research and Development Center for New Medical Frontiers, Kitasato University School of Medicine, Kanagawa, Japan; 19 Kitasato University School of Medicine, Kanagawa, Japan

**Keywords:** Biomarkers, Gastroenterology

## Abstract

Prostaglandin E-major urinary metabolite (PGE-MUM) is a urinary biomarker reflecting ulcerative colitis (UC) activity. This prospective observational study aimed to evaluate the usefulness of PGE-MUM via rapid chemiluminescent enzyme immunoassay in detecting endoscopic remission (ER) and histologic remission (HR) in pediatric UC (6–16 years) in comparison with fecal calprotectin (FCP). ER and HR were defined as Mayo endoscopic score (MES) of 0 and Matts’ histological grades (Matts) of 1 or 2, respectively. A total of 104 UC and 39 functional gastrointestinal disorder (FGID) were analyzed. PGE-MUM levels were significantly higher in the UC group than in the FGID group (*P* < 0.001). FCP levels were significantly elevated in the group without ER and HR than in the group with ER and HR (*P* < 0.001 and *P* = 0.001), whereas PGE-MUM levels were significantly higher in the group without ER compared to the group with ER (*P* < 0.001). No significant differences were noted in the AUCs for PGE-MUM and FCP in detecting ER and HR. Although PGE-MUM was inferior to FCP for the detection of HR, it might have the potential for application as a biomarker of endoscopic activity in pediatric UC owing to its noninvasive and rapid method.

## Introduction

In order to improve the prognosis of ulcerative colitis (UC), endoscopic mucosal healing has been proposed as the ultimate goal of UC treatment^1,2^. However, recent studies have highlighted the importance of histologic healing as a novel treatment target^3,4^. Colonoscopy coupled with biopsy is considered the gold standard for the monitoring endoscopic and histologic activities in patients with UC, regardless of age. Nonetheless, bowel preparation for colonoscopy has been perceived as the most difficult part of the procedure by both pediatric patients and their caregivers^5,6^. Additionally, in pediatric patients, sedation or general anesthesia is often necessary during colonoscopy, and this procedure is generally considered more invasive compared to adult patients^7^. Under these circumstances, several biomarker tests have been proposed as alternatives for colonoscopy. One such test is the fecal calprotectin (FCP) test, which is commonly used in UC to quantify calprotectin, a protein released from leukocytes, and its value reflects the degree of intestinal mucosal inflammation^8,9^. Blood-based biomarkers such as C-reactive protein (CRP), erythrocyte sedimentation rate (ESR), and leucine-rich α2-glycoprotein have been previously utilized to confirm healing in UC^10–12^. Although fecal- and blood-based biomarkers are useful, acquiring fecal or blood specimens in the pediatric filed may pose difficulties due to various concerns of patients or their guardians, such as pain, discomfort, timing, and messiness^13^. In recent years, the prostaglandin E-major urinary metabolite (PGE-MUM), that can be measured in urine, has been proposed as a biomarker^14^. PGE-MUM is a stable metabolite of prostaglandin E2 (an important chemical mediator of inflammation) and an indicator of large intestine inflammation in adult UC^15,16^. Moreover, we have also reported that PGE-MUM reflects endoscopic disease activity in pediatric UC^13^. Traditionally, PGE-MUM was measured using the radioimmunoassay (RIA) method; however, rapid measurement of PGE-MUM has recently become possible due to the development of fully automated chemiluminescent enzyme immunoassay (CLEIA)^17^.

Herein, we designed a multicenter prospective study to compare biomarkers, including PGE-MUM (measured via CLEIA) and FCP, in predicting endoscopic and histologic activity in pediatric UC.

## Results

### Patient characteristics

**Figure 1:**
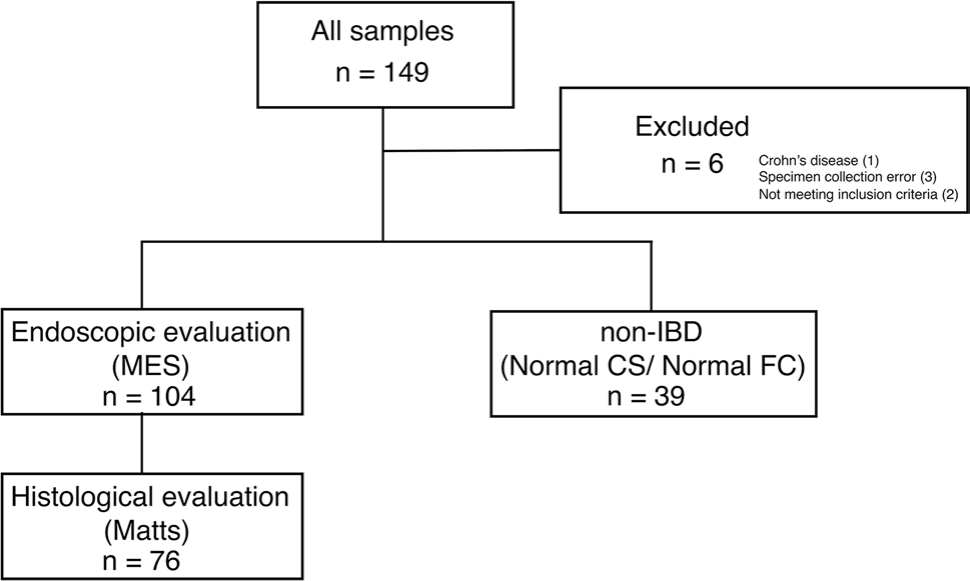
Flow chart depicting patient selection.

A total of 143 samples, including 104 UC patients and 39 patients with functional gastrointestinal disorders (FGIDs) as controls, were enrolled in this study from 14 pediatric centers in Japan between November 2018 and November 2020 (Fig. 1). After applying the inclusion and exclusion criteria, the final histological evaluation was performed in 76 UC patients because the remaining 28 tissue samples slides could not be sent due to changes in institutional regulations. The baseline characteristics of the patients with UC and controls are shown in Table 1. The PGE-MUM levels measured by the CLEIA in UC were significantly higher than those in controls (median 28.5 vs 18.6, *P* < 0.001).

**Table 1:** Patient characteristics.

	UC	Control
Total	104	39
Age (years), (mean ± SD)	12.6 ± 2.6	10.8 ± 2.9
Males, n (%)	51 (49%)	18(46%)
Disease extent		
E1 (proctitis)	7	
E2 (left-sided)	17	
E3 (extensive)	5	
E4 (pancolitis)	75	
Medications		
Aminosalicylate	71	
Corticosteroids	17	
Azathioprine/6-MP	32	
Biologic drugs	16	
Tacrolimus	1	
No medication	21	
PGE-MUM by CLEIA (μg g^−1^ Cr^−1^), median (IQR)	23.1 (16.1–38.6)	
All*	28.5 (16.2–46.3)	18.6 (15.7–22.6)
Male*	31.3 (17.0–47.9)	18.7(14.8–24.1)
Female*	26.0 (16.2–42.1)	18.6 (17–21.5)
PGE-MUM by RIA (μg g^−1^ Cr^−1^), median (IQR)	21.7 (14.9–34.9)	
FCP, median (IQR)	2480 (300–4830)	
CRP (mg/dl), median (IQR)	0.04 (0.02–0.12)	
ESR (mm), median (IQR)	11 (5–19)	
Clinical activity, PUCAI		
median (IQR)	20 (0–45)	
< 10/10–34/35–64/65 <	41/28/28/7	
Endoscopic activity, MES^†^ (104 subjects)		
Median (IQR)	2 (1–3)	
0/1/2/3, n	10/29/37/28	
Histologic activity, Matts^‡^ (76 subjects)		
Median (IQR)	4 (4–5)	
1/2/3/4/5, n	6/3/8/22/37	
Intervals of urine sampling and colonoscopy, day, mean (SD)	1.2 ± 2.0	
Intervals of fecal sampling and colonoscopy, day, mean (SD)	1.6 ± 3.2	

*CRP* C-reactive protein, *ESR* erythrocyte sedimentation rate, *IQR* interquartile range, *6-MP* 6-mercaptopurine, *MES* Mayo endoscopic score, *PGE-MUM* Prostaglandin E-major urinary metabolite, *PUCAI* Pediatric Ulcerative Colitis Activity Index, *UC* ulcerative colitis; *P < 0.001 ^†^Maximum MES among 6 segments (the cecum, ascending colon, transverse colon, descending colon, sigmoid colon, and rectum). ^‡^Maximum Matts among 6 segments.

### Correlation between PGE-MUM measured by CLEIA and RIA methods

**Figure 2:**
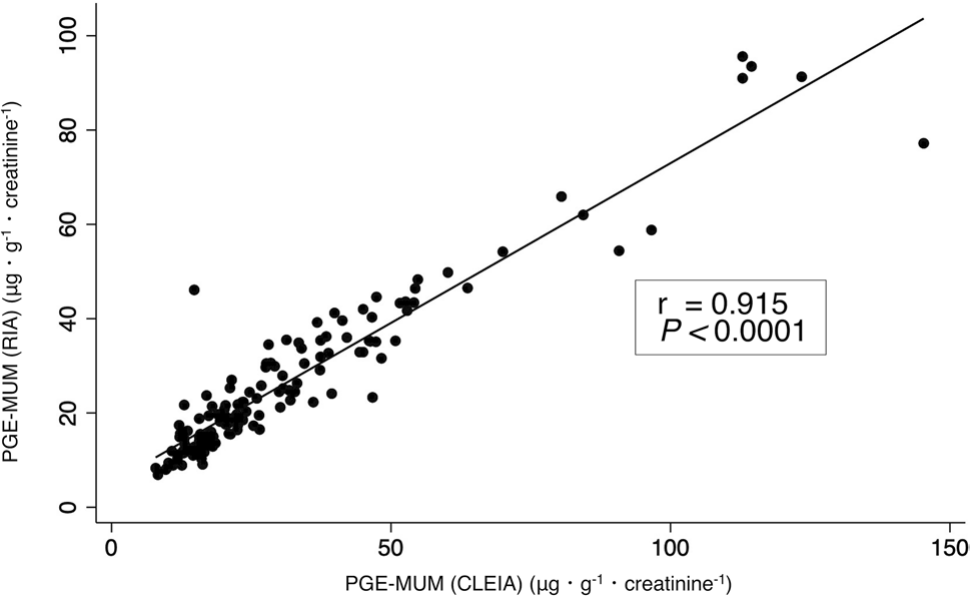
Spearman’s correlation among PGE-MUM levels was measured using both CLEIA and RIA.

In the analysis of the correlation between PGE-MUM levels measured using CLEIA and RIA, a strong correlation was found between PGE-MUM levels measured by both methods (Spearman’s rank correlation coefficient 0.915, *P* < 0.0001) (Fig. 2). The median values of PGE-MUM by CLEIA were statistically higher than the median values of PGE-MUM by RIA (Table 1).

### Correlations between biomarkers and clinical parameters (PUCAI, MES, Matts)

**Figure 3:**
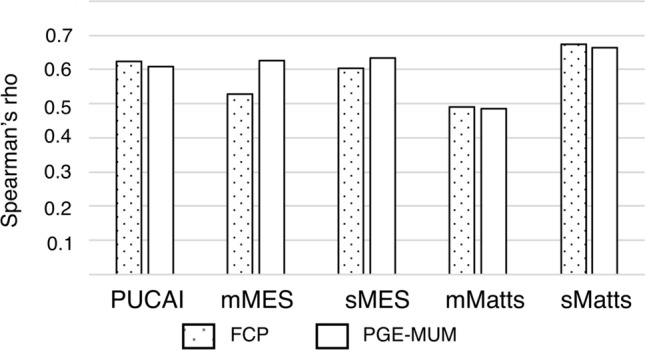
Spearman’s correlation among biomarkers (PGE-MUM, FCP) and evaluated parameters in pediatric UC. Y-axis indicates Spearman’s rho, and X-axis indicates all parameters (PUCAI, mMES, sMES, mMatts, and mMatts).

Analyses of the correlations between biomarkers (PGE-MUM, FCP) and clinical parameters (PUCAI, MES, and Matts) showed that PGE-MUM and FCP levels correlated well with each parameter compared to the other biomarkers (Fig. 3).

### Comparison between PGE-MUM and FCP levels by endoscopic activities (mMES and sMES)

**Figure 4:**
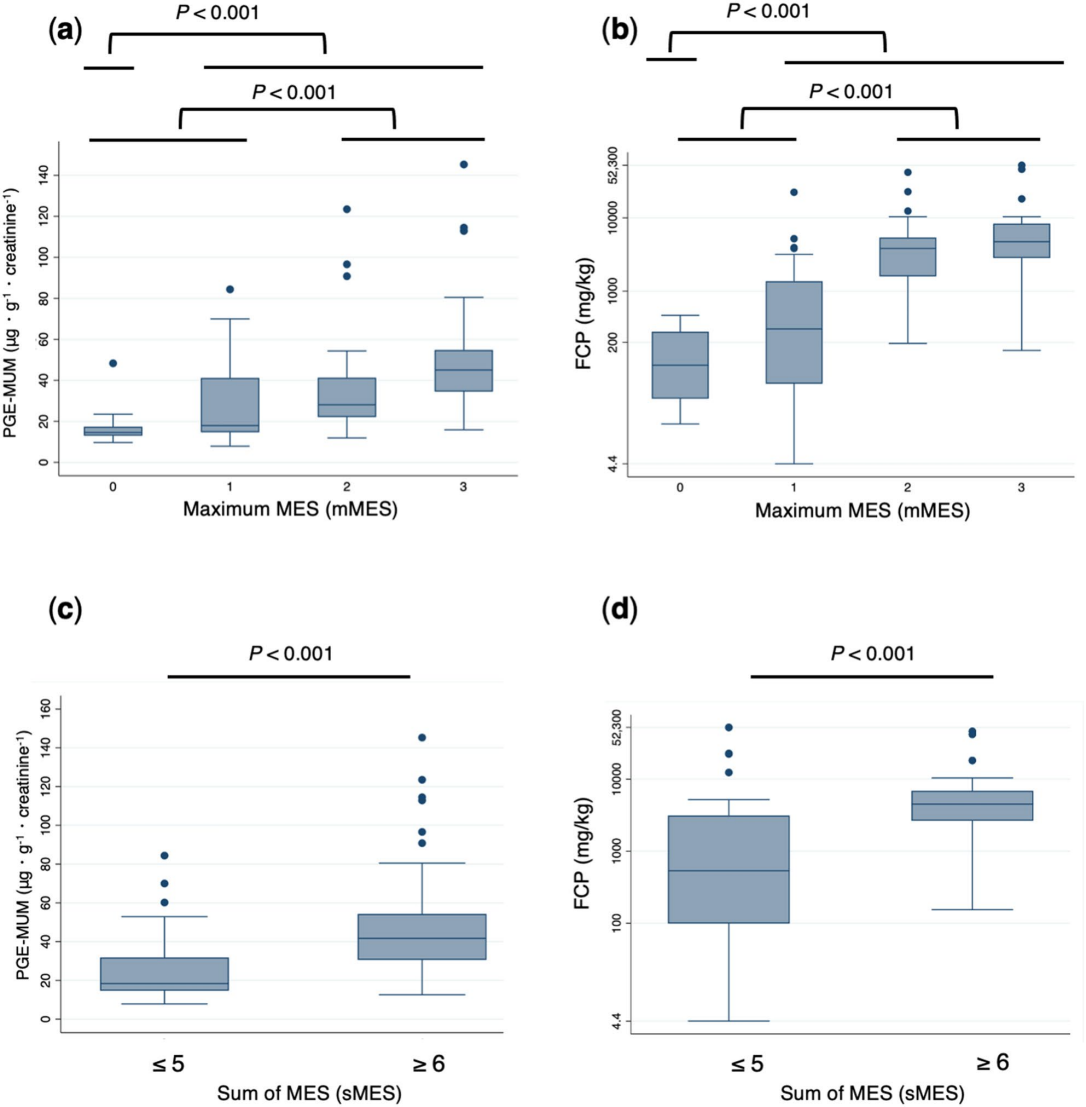
Comparisons of PGE-MUM and FCP levels for endoscopic disease activity (mMES and sMES) in UC patients. (**a**) The PGE-MUM, and (**b**) FCP values were significantly lower in the ER group (mMES 0) than the non-ER group (mMES 1–3); (**c**) PGE-MUM, and (**d**) FCP values were significantly lower in the group with sMES ≤ 5 than the group with sMES ≥ 6.

Correlations between the biomarkers and endoscopic findings (mMES) in the entire colon were analyzed. Both PGE-MUM and FCP levels were significantly elevated in patients with endoscopically active disease compared with the patients showing ER (Fig. 4a,b). As for endoscopic activities of the entire colon (sMES), both biomarker levels were significantly elevated in patients with sMES ≥ 6 compared with those who had sMES ≤ 5 (Fig. 4c,d).

### Comparison of PGE-MUM and FCP levels by histologic activities (mMatts and sMatts)

**Figure 5:**
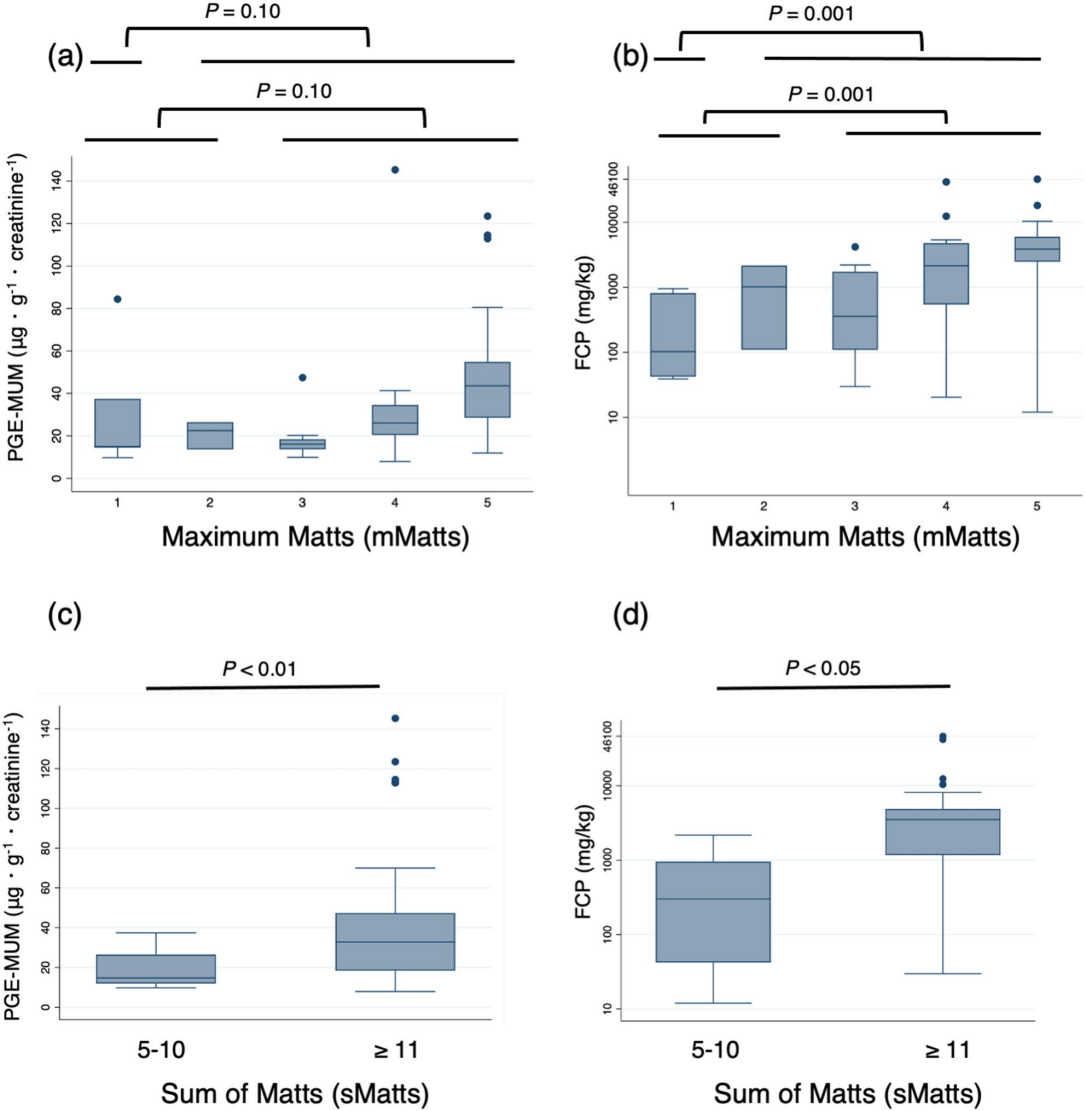
Comparisons of PGE-MUM and FCP levels for histologic activity (mMatts and sMatts) in UC patients. (**a**) PGE-MUM and (**b**) FCP values were significantly lower in the HR group (mMatts 1–2) than the non-HR group (mMatts 3–5); (**c**) PGE-MUM, and (**d**) FCP values were significantly lower in the group with sMatts between 5 and 10 than those in the group with sMatts ≥ 11.

Correlation analyses between biomarkers and histologic activities (mMatts) in the entire colon revealed that only the FCP levels were significantly elevated in patients with histologically active disease compared with those at HR (Fig. 5a,b).

As for histologic activities of the entire colon (sMatts), both biomarker levels were significantly elevated in patients with sMatts ≥ 11 compared with patients who had sMatts ≤ 10 (Fig. 5c,d).

### Predicting ER (MES 0 vs 1–3)

**Figure 6:**
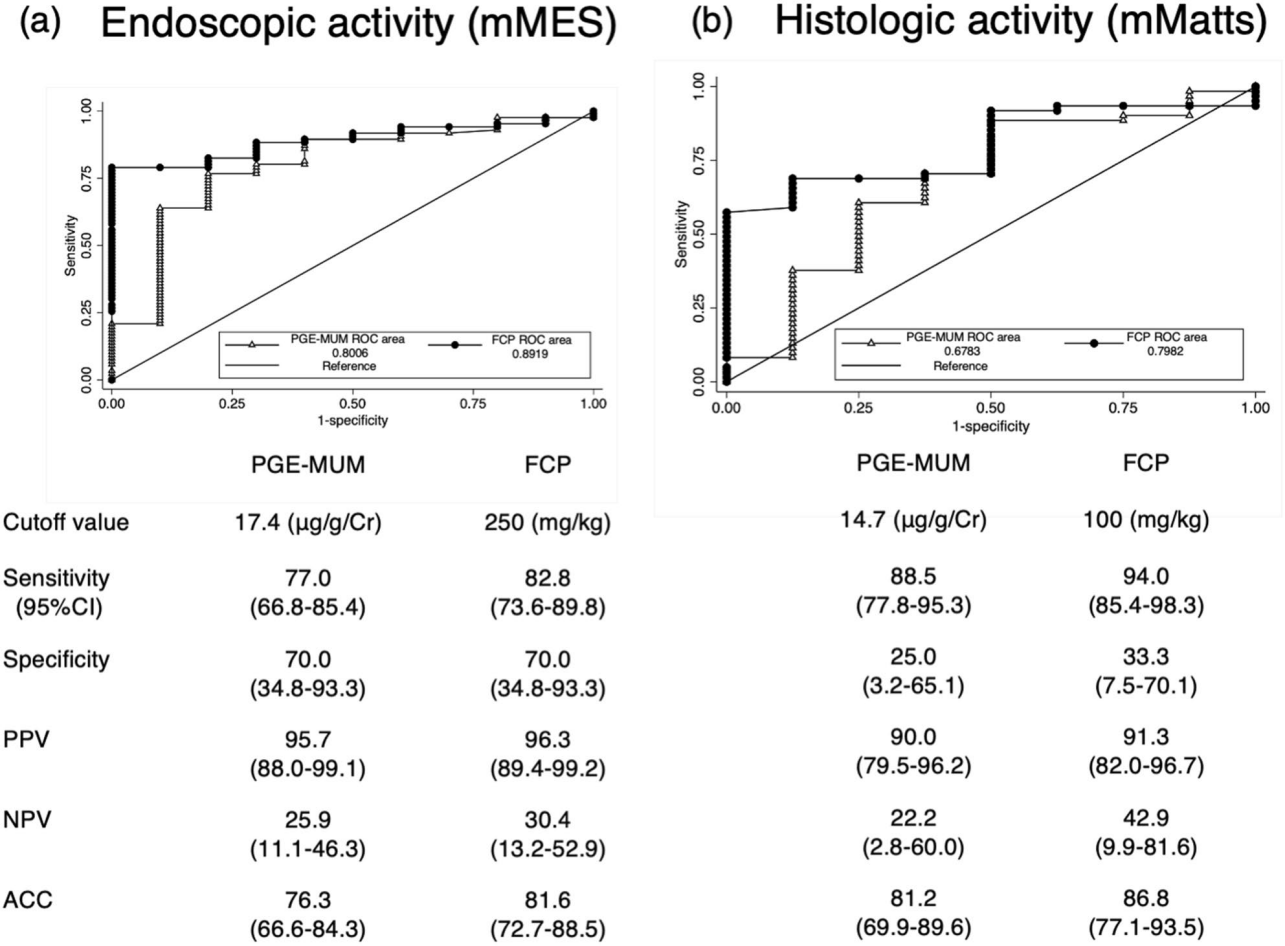
Receiver operating characteristic curve for PGE-MUM, FCP in relation to endoscopic (MES), and histologic activities (Matts) in UC patients. (**a**) mMES and (**b**) mMatts. Comparison of the area under the receiver operating characteristic (ROC) curves, optimal cutoff values, sensitivity, specificity, positive predictive value (PPV), negative predictive value (NPV), and accuracy (ACC) of PGE-MUM and FCP for the determination of endoscopic (**a**) and histologic (**b**) remission. The area under the ROC curves did not differ significantly.

ROC curve analysis showed that PGE-MUM and FCP had good predictive values for identifying patients with ER (AUC 0.80 and 0.89, respectively) (Fig. 6a). Although FCP showed a relatively high AUC, the difference was not statistically significant (*P* > 0.05). The cut-off value of PGE-MUM for predicting ER (mMES = 0) was 17.4 μg/g creatinine. Levels of PGE-MUM < 17.4 μg/g creatinine could discriminate ER from the absence of ER with a sensitivity of 0.77 and specificity of 0.70, whereas levels of FCP < 250 mg/kg could discriminate ER from the absence of ER with a sensitivity of 0.82 and specificity of 0.70 (Fig. 6a).

### Predicting pER (MES 0–1 vs 2–3)

ROC curve analysis showed that FCP had better predictive values for identifying patients with pER compared to PGE-MUM (AUC 0.87 and 0.74, respectively, *P* < 0.05) (Supplementary Fig. S1). The cut-off value of PGE-MUM for predicting ER (mMES = 0) was 20.4 μg/g creatinine. Levels of PGE-MUM < 20.4 μg/g creatinine could discriminate ER from the absence of pER with a sensitivity of 86.4 and specificity of 65.8, whereas levels of FCP < 250 mg/kg could discriminate pER from the absence of pER with a sensitivity of 95.3 and specificity of 51.3 (supplementary Fig. S1).

### Predicting HR

ROC curve analysis showed that FCP had a superior predictive value for identifying patients with HR (AUC 0.79) compared to PGE-MUM (AUC 0.67) (Fig. 6b). Although FCP showed a relatively high AUC, the difference was not statistically significant (*P* > 0.05). The cutoff value of PGE-MUM for predicting HR (mMatts = 1.2) was 14.7 μg/g creatinine. The cutoff value of PGE-MUM could discriminate HR from the absence of HR with a sensitivity of 0.88 and specificity of 0.25, whereas levels of FCP < 100 mg/kg could discriminate HR from the absence of HR with a sensitivity of 0.94 and specificity of 0.33 (Fig. 6b).

## Discussion

The present study is the first to report on the utility of rapidly measured PGE-MUM via CLEIA as a biomarker in pediatric UC. It is well-accepted that PGE2 is a crucial chemical mediator of inflammatory lesions, but the measurement of PGE2 is technically difficult because of its rapid metabolism^18^. PGE-MUM, a stable product of PGE2 metabolism, can be conveniently measured in urine samples. The stimulation of inflammatory cytokines, such as tumor necrosis factor-α, leads to the upregulation of cyclooxygenase-2, further leading to PGE2 secretion in inflamed colonic mucosal tissues of UC. In the blood, PGE2 is immediately metabolized to 15-keto-PGE2 by 15-hydroxyprostaglandin dehydrogenase (present in the lungs and colon). In the liver and kidney, 15-keto-PGE2 is converted into 13,14-dihydro-15-keto PGE2 by the action of prostaglandin Δ13-reductase, followed by β- and ω-oxidation, and is finally converted to PGE-MUM (7α-hydroxy-5,11-diketotetranor-prosta-1,16-dioic acid), which is excreted in the urine^19^. If the colon is highly inflamed, greater levels of PGE2 are secreted in the inflamed colonic mucosal tissue and released into the bloodstream, resulting in increased PGE-MUM levels in the urine. Arai et al. reported for the first time that PGE-MUM measured by RIA reflects the endoscopic (MES) and histologic (Matts grading) activity of UC in adult patients^15^. PGE-MUM is considered an ideal biomarker in pediatric UC patients because it can be measured in urine, which can be collected easily without causing pain. Furthermore, the technique of CLEIA has made it possible to measure PGE-MUM in a short time (less than 1 h)^17^.

In a previous study, we reported that PGE-MUM measured by RIA correlated with endoscopic and histologic activity in pediatric UC^13^. In this study, a good correlation was observed between PGE-MUM evaluated by CLEIA and the endoscopic and histologic activities of the entire colon in pediatric UC. Moreover, PGE-MUM had a diagnostic performance comparable to that of FCP for endoscopic activity; however, FCP had a superior diagnostic performance for detecting histologic activity.

Previous reports have proposed a multitude of threshold values for prognosticating ER and HR^15,16^. Sakurai et al. have suggested the employment of cutoff values of 14.5 and 14.2 µg/g･Cre to predict ER (mMES 0) and HR (mMatts 1–2), respectively^16^. Although the PGE-MUM values in their report were measured by RIA method, the pediatric and adult thresholds for PGE-MUM to predict ER may exhibit differences.

To the best of our knowledge, the present novel study is the largest prospective multicenter research on biomarkers in pediatric UC to date. Furthermore, the diagnostic performance of PGE-MUM in predicting ER (MES 0) was similar to that of FCP, which is accepted by Japanese health insurance, indicating that PGE-MUM can be sufficiently used as a biomarker for pediatric UC in routine practice.

In their study, Gupta et al*.* showed an association between higher relapse rates and persistent histologic activity in UC patients with ER^20^. Although there have been no reports on the usefulness of HR in pediatric UC, the achievement of HR in addition to ER might become an ultimate treatment goal in the future, as it can reduce clinical relapses in adult patients in whom HR has been achieved^3,4,20^. Biomarker studies on HR in UC are limited to the pediatric population. There have been some reports showing a strong correlation between FCP with histologic activity in pediatric UC, but the evidence has not been established because of the small sample sizes of these studies^21–23^. In our study, PGE-MUM was found to be inferior to FCP in terms of its diagnostic ability, slightly for ER but significantly for HR. Nonetheless, the ease of testing may outweigh the slightly inferior diagnostic utility, and more studies are needed in the future to better understand the role of PGE-MUM in pediatric UC.

Our study had several limitations. First, the target population for this study was 6–16 years old, so we could not evaluate whether PGE-MUM reflects disease activity in patients younger than six years, whose numbers have been increasing recently due to the emergence of early onset inflammatory bowel disease. Our unpublished data suggest that PGE-MUM levels are higher in infants; therefore, the normal value of PGE-MUM needs to be clarified, especially in infants. Furthermore, we did not use the Geboes score to assess histologic activity in our study, even though it has been validated as an accurate method of histologic assessment in UC patients^24,25^. In this study, FCP had better PPV regarding detecting HH. One of the reasons may be that the Matts grading weights inflammation cell infiltrations more heavily compared to the Geboes score. Future studies utilizing the Geboes scores in addition to Matts’ grading are needed to further clarify the relationship between various biomarkers and histologic disease activity in UC.

Overall, this study revealed that PGE-MUM was correlated with endoscopic activity in pediatric UC. Although it was somewhat inferior to FCP for HR prediction, it was almost identical to FCP in reflecting ER. PGE-MUM can be rapidly measured in urine by CLEIA, which is easier in terms of sample collection in comparison to stool or blood, and this is a clear advantage over FCP. Further studies are required before PGE-MUM can be used in clinical practice, including external validation as well as assessing reliability/agreement and responsiveness to changes after treatment.

## Methods

### Subjects and study design

Patients aged 6–16 years, who were evaluated and treated for UC at 14 participating Japanese pediatric centers between November 2018 and November 2020, were prospectively enrolled in this study. Additionally, controls diagnosed with FGIDs based on Rome IV by pediatric gastroenterologists were recruited from the Osaka Women’s and Children’s Hospital^26^. As the normal values for children remain elusive, FGID group was adopted to serve as a reference point. All controls underwent endoscopy including colonoscopy or FCP testing for diagnosis of FGIDs. UC patients aged between 6 and 16 years, who were diagnosed on the basis of the European Society for Pediatric Gastroenterology, Hepatology, and Nutrition Revised Porto Criteria, were included in this study^27^. The exclusion criteria were as follows: (1) presence of proteinuria; (2) history of diabetes; (3) current use of stimulant laxatives (sennoside)^28^, nonsteroidal anti-inflammatory drugs (NSAIDs)^29^; (4) history of colorectal surgery and (5) pulmonary disorders including chronic fibrosing interstitial pneumonia^30^. Samples (urine, stool, and blood) were collected within 2 weeks prior to colonoscopy because the laxatives used in colonoscopy preparation could influence the PGE-MUM levels^28^.

### Assessment of clinical, endoscopic, and histologic activity of UC

#### Clinical activity

The Pediatric Ulcerative Colitis Activity Index (PUCAI) was used to score the clinical activity of UC in each participant^31^. PUCAI was determined on the same day the samples were collected.

#### Endoscopic activity

The colonoscopic images taken in each institution were sent to Osaka Women’s and Children’s hospital. The assessment of endoscopic activity was determined using a blinded central review by three pediatric gastroenterologists. Any differences between these experts were resolved by consensus. Endoscopic activities were determined using Mayo endoscopic scoring (MES) for UC^32^. The colon was divided into five segments: cecum and ascending; transverse, descending, and sigmoid colon; and rectum. The highest endoscopic activity in the five segments was scored using the maximum MES (mMES). We defined the mMES of 0 as endoscopic remission (ER) and the MES 0–1 as partial endoscopic remission (pER). The sum of the maximum Mayo scores as modified Mayo scores (sMES) was calculated from the individual scores of the five segments, reflecting the endoscopic activity of the entire colon^13,33,34^*.*

#### Histologic activity

The histologic activity was determined using Matts’ histologic grading system^35^. Biopsy samples were collected from the most active inflammatory tissue in each segment of the colon under colonoscopic observation^13^. The biopsy samples were analyzed blindly by two pathologists. Any differences between two experts were resolved by consensus. Although ER was observed in the colon, mucosal specimens were obtained randomly from each segment. The most severe instance of inflammation in each patient was determined using the Matts score (mMatts). We defined mMatts of 1 and 2 as histologic remission (HR). The sum of the Matts scores as modified Matts scores (sMatts) was calculated from the individual scores of the five segments, reflecting the histologic activity of the entire colon^13,16^.

### Measurement of biomarkers

#### PGE-MUM analysis

Spot urine samples were obtained before the day of the colonoscopy and centrifuged at 1000×*g* for 10 min. The derived supernatants were then stored at − 30 °C until analysis. All urine samples were sent to Fujirebio (Tokyo, Japan), where they were measured using both CLEIA and RIA methods, as previously described^13,14,16,17,19^. In CLEIA methods, PGE-MUM levels in urine samples were measured using a one-step immunoassay method based on CLEIA technology on the LUMIPULSE System (Fujirebio Inc., Tokyo, Japan). Prior to the immunoassay, the urine samples were treated with an alkaline solution to transform the PGE-MUM in the samples into bicyclic PGE-MUM, which is the form detected by the immunoassay. The bicyclic PGE-MUM in specimens and on particles competitively react with alkaline phosphatase-labeled anti-bicyclic PGE-MUM monoclonal antibodies, and antigen–antibody immunocomplexes are formed. After washing, a luminescent substrate solution is added, and the luminescent signal is measured. The PGE-MUM concentration of a specimen is calculated from the calibration curve^17^. In RIA methods, PGE-MUM levels in urine samples were measured using a radioimmunoassay kit (Institute of Isotopes Co., Ltd, Budapest, Hungary)^19^. Alkaline treatment was conducted by adding 100 μL of 1-mol NaOH to 50 μL of the urine sample. Thereafter, the samples were stored at room temperature for 30 min. With this treatment, the PGE-MUM in the sample was converted to bicyclic PGE-MUM. After neutralization by adding 100 μL of 1-mol hydrochloric acid, the treated urine sample was further diluted fivefold with 1000 μL of assay buffer (50-mM phosphate buffer, pH 7.4, containing 0.1% gelatin and 0.1% sodium azide). A sample or standard (100 μL) was dispensed into a reaction tube, and 100 μL of 125 I-bicyclic PGE-MUM (approximately 680 Becquerel) and 100 μL of rabbit antiserum to bicyclic PGE-MUM were added. After overnight incubation at 2–8 °C, 250 μL of the separating agent containing paramagnetic particles coated with antirabbit immunoglobulin was added to each tube and was incubated for 15 min at room temperature. The bound fraction was separated by centrifugation, and the radioactivity of each tube was measured^16^. The measured PGE-MUM values (CLEIA and RIA) were corrected for urinary creatinine levels.

#### FCP analysis

Fecal samples for measuring FCP concentration were collected within two weeks before the day of the colonoscopy. The samples were stored at − 20 °C for subsequent measurements. FCP levels were determined using the EliA Calprotectin 2 kit (Thermo Fisher Scientific, Tokyo, Japan).

### Statistical analysis

For data analysis, continuous variables were expressed as the median and interquartile range (IQR). The Spearman’s rank correlation was used to investigate the relationships between the studied biomarkers. The Wilcoxon rank-sum test was applied to compare the groups. A receiver operating characteristic (ROC) curve was used to determine the cutoff value of each biomarker to predict ER or HR in patients with UC, and the corresponding area under the ROC curve (AUC) was also calculated. Further, to evaluate the cutoff points of PGE-MUM, we used the Youden index^36^. The cutoffs for ER and HR, as determined by FCP analysis (< 250 mg/kg and < 100 mg/kg, respectively), were adapted as the standard cutoffs^37,38^. Thereafter, we examined the sensitivity, specificity, predictive values, and accuracy of all evaluated biomarkers for ER and HR. All statistical analyses were performed using STATA 17.0 (StataCorp, College Station, TX, USA) and EZR (Saitama Medical Center, Jichi Medical University, Saitama, Japan), which is a graphical user interface for R (The R Foundation for Statistical Computing, Vienna, Austria). *P* values of < 0.05 were considered statistically significant.

### Ethical statement

The study protocol complied with the ethical guidelines stated in the Declaration of Helsinki (2013 revision) and was approved by the Ethics Committee of Osaka Women’s and Children’s Hospital (registration number 1157-8) and its counterparts at other participating centers. Written informed consent was obtained from all enrolled patients and/or their parents.

## Supplementary Information

Supplementary Figure S1.

Supplementary Legends.

## Data Availability

The data underlying this article will be shared by the corresponding author on reasonable request.
