# Supplementary figures and images for: Utility of a rapid assay for prostaglandin E-major urinary metabolite as a biomarker in pediatric ulcerative colitis
Source: Sci Rep. 2023 Jun 19;13:9898. doi: 10.1038/s41598-023-37145-6 (PMC10279732; doi:10.1038/s41598-023-37145-6)

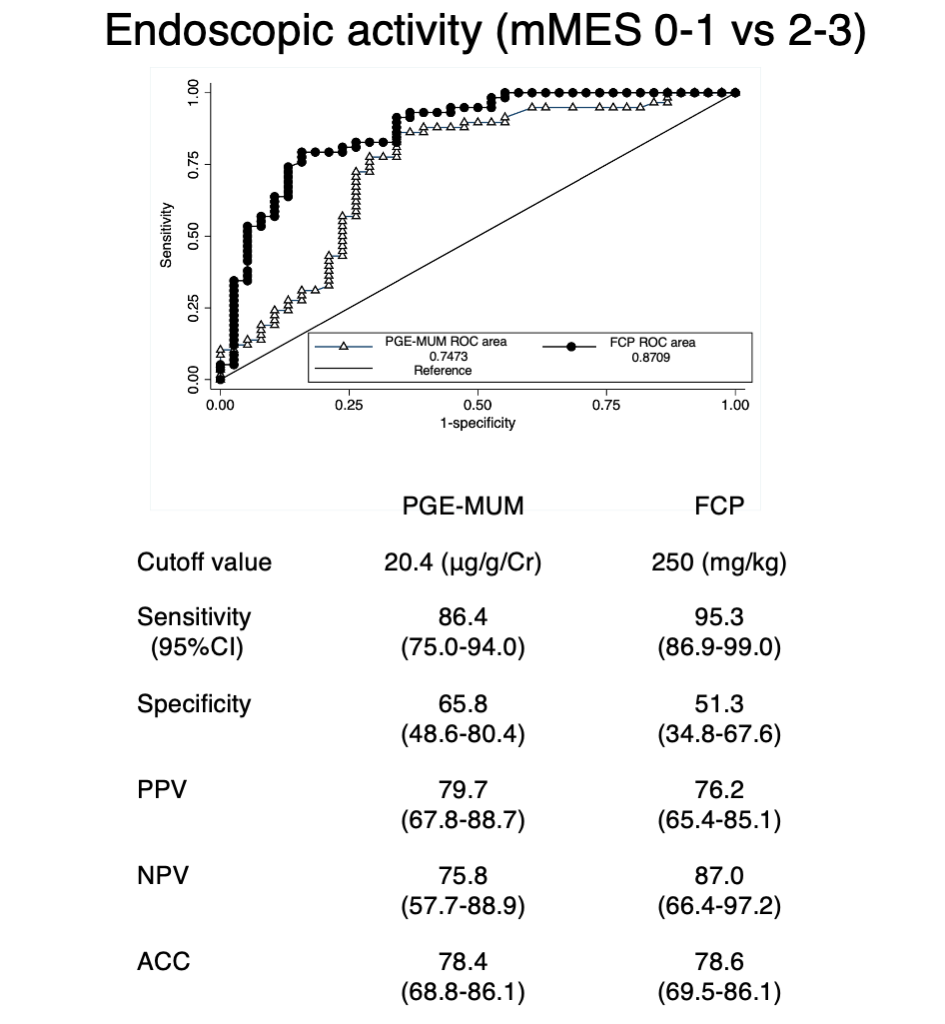

Supplement: Supplementary file 1 — Supplementary Figure S1. [file 41598_2023_37145_MOESM1_ESM.tiff]
