# Supplementary material for: Utility of a rapid assay for prostaglandin E-major urinary metabolite as a biomarker in pediatric ulcerative colitis
Source: Sci Rep. 2023 Jun 19;13:9898. doi: 10.1038/s41598-023-37145-6 (PMC10279732; doi:10.1038/s41598-023-37145-6)
Supplement: Supplementary file 2 — Supplementary Legends. [file 41598_2023_37145_MOESM2_ESM.docx]

Supplementary Figure. S1 Receiver operating characteristic curve for PGE-MUM, FCP in relation to endoscopic (MES) in UC patients. Comparison of the area under the receiver operating characteristic (ROC) curves, optimal cutoff values, sensitivity, specificity, positive predictive value (PPV), negative predictive value (NPV), and accuracy (ACC) of PGE-MUM and FCP for the determination of partial endoscopic remission (mMES 0-1). The area under the ROC curves achieved by FCP was significantly higher than that by PGE-MUM (*P* < 0.05).
